# Kidney Disease in Disadvantaged Populations

**DOI:** 10.1155/2012/427589

**Published:** 2012-12-31

**Authors:** Rudolph A. Rodriguez, Li-Li Hsiao, J. Kevin Tucker, David Pugsley

**Affiliations:** ^1^Department of Medicine, VA Puget Sound Healthcare System and University of Washington, Seattle, WA 98108, USA; ^2^Renal Division, Brigham and Women's Hospital, Boston, MA 02115, USA; ^3^Department of Nephrology and Transplantation, The University of Adelaide, Adelaide, SA 5005, Australia

The challenges and dilemmas faced by disadvantaged populations with kidney disease are tremendous. Patients, health care providers, and investigators struggle to manage and understand the high burden of disease, the practical approach to delivery of high cost renal replacement therapy with limited resources, implementation of preventive strategies, and the difficult ethical dilemmas. The purpose of this special issue is to shed light on the understudied challenge of kidney disease in disadvantaged populations.

The papers in this special issue serve as an example of the excellent research conducted in developing countries, many times in collaboration with investigators in developed countries. Coincidentally, the majority of papers include authors from Nigeria, a country which likely is representative of sub-Saharan Africa. In editing this special issue, the barriers faced by investigators from developing countries were evident and include lack of funding, limited forums to disseminate their work, the peer review process, and lack of resources. Along with a topical review article “*Chronic Kidney Disease in Disadvantaged Populations*,” the papers in this issue provide an overview of some of the important issues in sub-Saharan Africa including new biomarkers for kidney disease, “*Circulating Adiponectin Is Associated with Renal Function Independent of Age and Serum Lipids in West Africans*”; association between APOL1 and HDLc, “*Variation in APOL1 Contributes to Ancestry-Level Differences in HDLc-Kidney Function Association*”; early markers of kidney disease, “*Prevalence and Correlates of Microalbuminuria in Children with Sickle Cell Anaemia: Experience in a Tertiary Health Facility in Enugu, Nigeria*” and “*Association between Urinary N-Acetyl-Beta-D-Glucosaminidase and Microalbuminuria in Diabetic Black Africans*”; alternatives to high cost erythropoiesis-stimulating agents, “*Meta-Analysis of Randomized Controlled Trials on Androgens versus Erythropoietin for Anaemia of Chronic Kidney Disease: Implications for Developing Countries*”; and the disturbing issue of how to best provide care to patients with end-stage renal disease (ESRD), “*A Single-Center 7-Year Experience with End-Stage Renal Disease Care in Nigeria—A Surrogate for the Poor State of ESRD Care in Nigeria and Other Sub-Saharan African Countries: Advocacy for a Global Fund for ESRD Care Program in Sub-Saharan African Countries*.”

In many countries, dialysis is not an option for patients with ESRD. According to Dr. Saraladevi Naicker, the burden of kidney disease is thought to be high in sub-Saharan Africa, but the dialysis treatment rate ranges from 70 patients per million population in South Africa to 20 per million population in most of sub-Saharan Africa [1]. These numbers can be compared to the prevalence of ESRD in the United States among African-Americans which according to 2010 United States Renal Data Systems data was 5,242 per million population [2]. The difference in treatment rates provides a rough estimate of the number of people dying from ESRD without treatment in sub-Saharan Africa. Some medical centers in sub-Saharan Africa offer chronic hemodialysis to patients who can afford to pay. Given the extent of poverty in sub-Saharan Africa, it is not surprising that many patients with ESRD are provided dialysis for a few days to weeks and once the patient and their families deplete their funds, dialysis is discontinued [3] (also see, “*A Single-Center 7-Year Experience with End-Stage Renal Disease Care in Nigeria—A Surrogate for the Poor State of ESRD Care in Nigeria and Other Sub-Saharan African Countries: Advocacy for a Global Fund for ESRD Care Program in Sub-Saharan African Countries*”). In patients with true ESRD with no hope of renal recovery, is it ethical to provide dialysis for a few days or weeks depending on the funds available to the patient? Should dialysis be offered only to those with acute kidney injury and some hope of renal recovery? In a part of the world where per capita expenditure on health care is so low, how should dialysis be offered in an equitable and fair manner? How should patients with ESRD be managed humanely and with dignity?

What happens to patients with ESRD when dialysis is not an option? “The patients are discharged and then they disappear” is, unfortunately, a common answer to this question. One of the papers in this special issue describes the decisions faced by nephrologists in Nigeria which must mirror the same decisions faced in many countries in Africa, Asia, and Central America, (see “*A Single-Center 7-Year Experience with End-Stage Renal Disease Care in Nigeria—A Surrogate for the Poor State of ESRD Care in Nigeria and Other Sub-Saharan African Countries: Advocacy for a Global Fund for ESRD Care Program in Sub-Saharan African Countries*”). The ethical dilemmas are similar those faced by nephrologists in the United States in the 1960's when dialysis was in its infancy. Of course, patients do not disappear but instead die from uremia which is often mistakenly described as a “good” way to die. In the United Kingdom and the United States, the importance of palliative care for ESRD patients is now being recognized and promoted [4–6]. Many countries now recognize that the burgeoning population of elderly patients with a large number of comorbidities may not benefit from dialysis and may live just as long with a conservative approach focused on palliation of symptoms. In many countries, dialysis is not offered to young patients with ESRD. The cost of dialysis may be prohibitive in a country like Nigeria but palliative care should not be forgotten and should be considered as the ethical approach to care for these patients. Of course, most important are efforts to identify preventive strategies and early detection of chronic kidney disease along with novel approaches of care in disadvantaged populations.

The guest editors hope that the readership of International Journal of Nephrology finds this issue informative and thought provoking.

*Rudolph A. Rodriguez* *Li-Li Hsiao* *J. Kevin Tucker* *David Pugsley*
